# PFAS Exposure is Associated with a Lower Spermatic Quality in an Arctic Seabird

**DOI:** 10.1021/acs.est.4c04495

**Published:** 2024-10-23

**Authors:** Ségolène Humann-Guilleminot, Pierre Blévin, Geir Wing Gabrielsen, Dorte Herzke, Vladimir A. Nikiforov, William Jouanneau, Børge Moe, Charline Parenteau, Fabrice Helfenstein, Olivier Chastel

**Affiliations:** † Department of Environmental Science, Radboud Institute for Biological and Environmental Sciences (RIBES), Faculty of Science, Radboud University, Nijmegen 6500, the Netherlands; ‡ Laboratory of Evolutionary Ecophysiology, Institute of Biology, University of Neuchâtel, Neuchâtel 2000, Switzerland; § Centre d’Etudes Biologiques de Chizé, UMR 7372 CNRS - Université de La Rochelle, Villiers-en-Bois 79360, France; ∥ Akvaplan niva AS, Fram Centre, Tromsø NO-9296, Norway; ⊥ Norwegian Polar Institute, Fram Centre, Tromsø NO-9296, Norway; # Norwegian Institute for Air Research, Fram Centre, Tromsø NO-9296, Norway; ∇ Norwegian Institute for Nature Research, Trondheim NO-7034, Norway; ○ Department of Clinical Research, University of Bern, Bern 3010, Switzerland

**Keywords:** black-legged kittiwake, per- and polyfluoroalkyl substances, sperm morphology, sperm velocity, testosterone, luteinizing hormone, corticosterone, svalbard

## Abstract

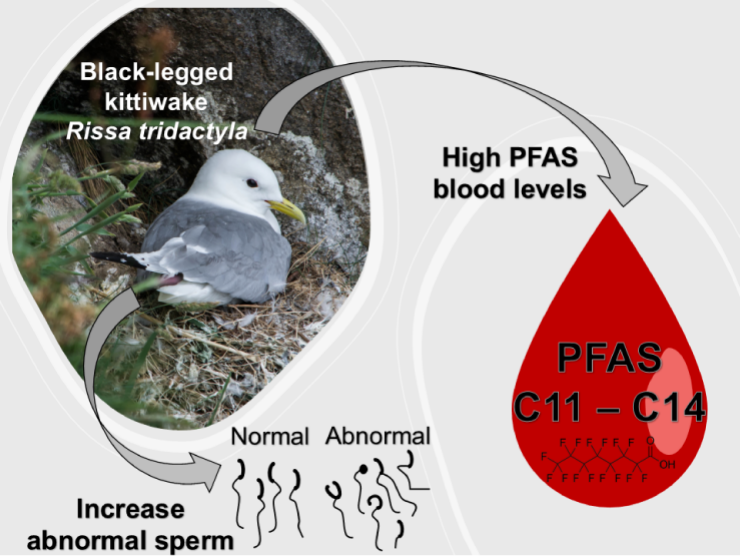

Several studies have reported an increasing occurrence of poly- and perfluorinated alkyl substances (PFASs) in Arctic wildlife tissues, raising concerns due to their resistance to degradation. While some research has explored PFAS’s physiological effects on birds, their impact on reproductive functions, particularly sperm quality, remains underexplored. This study aims to assess (1) potential association between PFAS concentrations in blood and sperm quality in black-legged kittiwakes (*Rissa tridactyla*), focusing on the percentage of abnormal spermatozoa, sperm velocity, percentage of sperm motility, and morphology; and (2) examine the association of plasma levels of testosterone, corticosterone, and luteinizing hormone with both PFAS concentrations and sperm quality parameters to assess possible endocrine disrupting pathways. Our findings reveal a positive correlation between the concentration of longer-chain perfluoroalkyl carboxylates (PFCA; C11–C14) in blood and the percentage of abnormal sperm in kittiwakes. Additionally, we observed that two other PFAS (i.e., PFOSlin and PFNA), distinct from those associated with sperm abnormalities, were positively correlated with the stress hormone corticosterone. These findings emphasize the potentially harmful substance-specific effects of long-chain PFCAs on seabirds and the need for further research into the impact of pollutants on sperm quality as a potential additional detrimental effect on birds.

## Abstract 2

This research investigates, for the first time, the correlation between PFAS concentrations in blood and sperm quality in Arctic black-legged kittiwakes, revealing an increase in the percentage of abnormal spermatozoa as PFAS blood concentrations rise.

## 1 Introduction

Per- and polyfluoroalkyl substances (PFASs) constitute a large family of synthetic chemicals, massively used for their unique surfactant properties, and characterized by their strong carbon–fluorine bonds that confer resistance to environmental and biological degradation.^1^ This resilience contributes to the pervasive presence of PFASs in ecosystems globally, with occurrence in water, soil, atmosphere and living organisms, raising concerns about their potential for bioaccumulation and the associated environmental and health risks.^2−4^ Over the last few decades, there has been a significant increase in research documenting the accumulation and effects of PFASs in wildlife. Seabirds, for instance, have been shown to bioaccumulate PFASs, as indicated by the measurement of relatively high concentrations of several perfluoroalkyl carboxylates (PFCAs) and perfluoroalkanesulfonates (PFSAs) as well as emerging or alternative PFAS in blood, eggs, and internal organs.^5−9^ Some PFASs are shown to be endocrine and metabolic disruptors, with the potential to alter key physiological functions, and consequently influence male reproductive capabilities through alteration of the hormonal balance.^4,7^ For instance, testosterone and luteinizing hormone (LH)—the primary hormone that stimulates testosterone production—are both major hormones involved in physiological and behavioral processes that enhance fecundity in vertebrates,^4,10,11^ Notably, studies on domestic geese have shown that LH and testosterone are linked to sperm quality, including sperm count, motility, and morphology. A decline in these hormones is associated with the progressive regression of efficiency of spermatogenesis.^12^ In contrast, increases in LH and testosterone have been associated with enhanced sperm motility and a higher percentage of normal sperm morphology in zebra finches and Northern pintails.^13,14^ Corticosterone is known for its wide-ranging effects on metabolism and behavior but has also been linked to reproductive function.^15^ Notably, research indicates that elevated level may negatively affect testis development and sperm motility in roosters.^16−18^ Therefore, disruptions in the equilibrium between these hormones could potentially lead to compromised spermatogenesis and overall reproductive capacity.

In black-legged kittiwakes (*Rissa tridactyla*), sentinels of Arctic environmental pollutants, PFAS exposure is linked to several physiological disruptions. These include alterations in carotenoid-based colorations, hinting at carotenoid metabolism interference,^19^ elevated thyroid hormones leading to increased energy expenditure^20,21^ and changes in oxidative stress markers alongside reduced corticosterone levels, suggesting stress response and metabolic impacts.^7,22^ Previous research on the reproductive effects of PFAS has been limited and yields contradictory findings. While some studies have identified an impact of PFAS on hatching success, others have not observed any significant reproductive effect.^23^ In addition, research across various bird species has highlighted how pollutants, such as pesticides and heavy metals, can detrimentally affect sperm quality and reproductive functions.^24−26^ However, to the best of our knowledge, the specific impact of PFASs on sperm quality in wild birds remains unexplored.

In a Svalbard-nesting population of black-legged kittiwakes (*Rissa tridactyla*) that are highly exposed to PFAS,^7^ we recently established a noninvasive method to collect viable sperm samples based on a simple massage technique applied to male kittiwakes, which exhibited a high proportion of abnormal sperm cells.^27^ This opens new research opportunities to accrue evidence of PFAS toxicity and document any possible consequences on reproductive function in seabirds. In this context, this study aims to (1) examine the relationships between plasma PFAS concentrations in black-legged kittiwakes and sperm quality parameters, including the proportion of abnormal spermatozoa, sperm velocity, percentage of motile spermatozoa and intraindividual variation in sperm size; and (2) examine the association of plasma levels of hormones involved in spermatogenesis (testosterone, corticosterone, and luteinizing hormone,^10,28^ with both PFAS concentrations and sperm quality parameters to assess possible endocrine disrupting pathways. The choice of these sperm quality parameters relies on several variables that, individually or in combination, have been demonstrated to effectively predict the fertilizing potential of sperm across various species.^29,30^ Compared to other species, male kittiwakes exhibit a substantial extent of intramale, within-ejaculate variation in sperm morphology.^27^ Several nonmutually exclusive explanations as to why sperm morphology varies within ejaculates despite stabilizing selection in favor of an optimal sperm design have been put forward.^31−33^ In kittiwakes, a species with low levels of sperm competition if any, the most likely explanation for intramale, within-ejaculate variation in sperm morphology is the production of large sperm numbers associated with errors in sperm production due to inevitable developmental errors. Therefore, we hypothesized that exposure to PFASs may disturb sperm quality control processes and lead to higher levels of within-ejaculate variation in sperm morphology.

We hypothesized that PFAS concentrations will negatively correlate with sperm velocity (VCL) and the percentage of motile sperm, while we expected positive correlations with the percentage of abnormal spermatozoa and within-ejaculate variance in morphology, suggesting an alteration of the sperm quality control at higher PFAS exposures. We anticipated that PFAS levels would be associated with lower testosterone and LH levels, while corticosterone levels may rise with PFAS exposure, suggesting potential hormonal disruption and stress-related endocrine effects. We predict that lower testosterone and LH levels would correlate with reduced sperm motility along with increased abnormalities and variance in sperm morphology, reflecting their role in sperm quality control. Conversely, elevated corticosterone levels could be associated with reduced sperm motility and increased abnormalities and within-ejaculate variance.

## 2 Material and Methods

### 2.1 Field Study Design

Fieldwork was conducted from May 25th to June sixth, 2016, and from May 25th to June third, 2017, in a black-legged kittiwake colony at Kongsfjord (Krykkjefjellet, 78°54′N, 12°13′E), Svalbard (European Arctic). We investigated a total of 76 males (*n* = 50 in 2016 and *n* = 26 in 2017), capturing them at their nests with a nylon noose attached to a telescopic pole during the prelaying phase, which includes both courtship and copulation. However, we only analyzed a subset of males to match the sample size of hormones and/or sperm quality parameters samples (*n* = 25 in 2016 and *n* = 26 in 2017; Table S1). Males previously identified through molecular sexing techniques^34^ were recognized by white PVC bands, marked with a three-letter code, and attached to their tarsus. We monitored the nests of sampled birds every 2 days with a mirror on a telescopic pole to determine the laying date.

### 2.2 Blood and Sperm Collection

After capture, we collected a blood sample (approximately 0.5 mL) from the brachial vein within 3 min using a heparinized syringe and a 25-gauge needle. We considered blood samples taken within this 3 min to reflect baseline corticosterone levels because there was no statistical evidence of a relationship between handling time and corticosterone levels (*p* = 0.16).^35^ Another blood sample (approximately 2 mL) was collected to measure the PFAS concentrations. These samples were immediately chilled on ice in the field and, upon return to the lab, separated into plasma and red blood cells (RBCs) by centrifugation and then stored at −80 °C for hormones or at −20 °C for PFASs analyses.

Sperm samples were successfully collected from a subset of 43 males (*n* = 17 in 2016 and *n* = 26 in 2017) by gently massaging the lower back and base of the tail, following the protocol described by Humann-Guilleminot and colleagues.^27^ Approximately 5 μL of sperm was mixed with 5 μL of DMEM (Dulbecco’s modified Eagle medium, 4500 mg glucose/L, 110 mg/L sodium pyruvate, and l-glutamine) heated at 40 °C (average body temperature of kittiwakes; Barrett 1978; Brent et al. 1983) and 3 μL of the mix sperm–DMEM were transferred into a 20 μm deep chamber slide (Leja Products B.V., The Netherlands) for video recording under the microscope (Olympus BX43 microscope – Olympus Co., Japan—with a 10× objective under negative phase contrast). We maintained the mix sperm–DMEM at 40 °C using a heating glass plate fitted to the microscope stage (MATS-U55S, Olympus Co., Japan). A small droplet from the ejaculate was also immediately smeared with 10% formalin (1:9 v:v; i.e., 4% formaldehyde) on a glass slide.

### 2.3 Sperm Quality Parameters

We assessed sperm quality following protocol described in Humann-Guilleminot et al., 2018.^27^ In brief, we recorded 5-s videos on four to five different fields to maximize the number of tracked spermatozoa. From each video, we used the computer-assisted sperm analysis (CASA) plugin^36^ for ImageJ^37^ to estimate curvilinear velocity (VCL: total point-to-point distance traveled by the sperm over the time period analyzed averaged to a per second value, μm/s) in samples collected in 2016 and 2017. The CASA also estimated the percentage of motile sperm.

In 2016 only, from each slide with the mix sperm-formalin, we took photos of ten intact sperm cells using the Nikon ACT-1 v2.70 software (Nikon Corporation, Japan) with a Nikon DFC7000T camera (Nikon Corporation, Japan) mounted on a Leica DMR microscope (Leica Microsystems GmbH, Germany) at 400× magnification and phase contrast 2. About 7 to 16 sperm cells (mean ± SE: 10.1 ± 0.5) per ejaculate were measured for head, midpiece, flagellum, and total length. Additionally, each cell was independently measured twice to assess the amount of variance due to measurement error using random models.^38^ The percentage of measurement error was 4.7% for head length, 12.5% for midpiece length, 1.3% for flagellum length, and 0.2% for total length. The average coefficient of variation [(SD/mean) × 100] for the two measures of the same sperm was 2.5% for head length, 5.2% for midpiece length, 1.1% for flagellum length, and 0.6% for total length. For further analyses, intramale variation in sperm length was assessed by calculating the standard deviation of the mean total sperm length.

In 2016 and 2017, sperm smears were also used to assess the percentage of abnormal sperm based on 50 spermatozoa per slide randomly selected. Spermatozoa were classified as morphologically normal, with abnormal head (no head, S-shaped head, bended head, no acrosome, burst head), with abnormal midpiece (no midpiece, broken midpiece) or with abnormal flagellum (no flagellum, broken flagellum, folded flagellum, flagellum with 90° angle, coiled flagellum, double flagellum, split flagellum) as described in Humann-Guilleminot et al., 2018.^27^

### 2.4 Hormones

Testosterone, corticosterone, and luteinizing hormone analyses were performed using radioimmunoassay at the Centre d’Etudes Biologiques de Chizé (CEBC), France and following the protocols and methods detailed in Goutte et al., 2010.^39^ We measured baseline levels of corticosterone, testosterone, and luteinizing hormone (LH) in plasma samples collected in 2016 only. Nine concentrations of testosterone were below the limit of detection (i.e., 0.45 ng/mL). Therefore, we substituted the values that were below the LOD following the robust regression on order statistics (ROS) method.^40^

### 2.5 PFASs

PFASs concentrations were determined from plasma of 76 male kittiwakes (*n* = 50 in 2016; *n* = 26 in 2017) at the Norwegian Institute for Air Research (NILU) in Tromsø, Norway. We searched for 20 compounds: perfluorooctanesulfonamide (PFOSA), perfluorobutanesulfonate (PFBS), perfluoropropanesulfonate (PFPS), perfluorohexanesulfonate (PFHxS), perfluoroheptanesulfonate (PFHpS), linear and branched perfluorooctanesulfonate (PFOS), perfluorononanesulfonate (PFNS), pefluorodecanesulfonate (PFDcS), perfluorohexanoate (PFHxA), perfluoroheptanoate (PFHpA), perfluorooctanoate (PFOA), perfluorononanoate (PFNA), perfluorodecanoate (PFDcA), perfluoroundecanoate (PFUnA), perfluorododecanoate (PFDoA), perfluorotridecanoate (PFTrA), and perfluorotetradecanoate (PFTeA), and two precursors, the fluorotelomer sulfonates (6:2 and 8:2 FTS). Only individual compounds detected in at least 70% of the samples were kept for further analyses (i.e., 1 PFSA: PFOSlin, and 6 PFCAs: PFNA, PFDcA, PFUnA, PFDoA, PFTrA and PFTeA). In addition, we also assessed the total plasma concentration of all detected PFASs subgroup PFCAs (PFNA, PFDcA, PFUnA, PFDoA, PFTrA and PFTeA; hereafter termed ∑PFCAs) and the total plasma concentration of all detected PFASs (PFCAs and PFOSlin; hereafter referred as ∑PFASs). Only PFTeA contained values below the LOD and we substituted these values following the robust regression on order statistics (ROS) method.^40^ The detailed methodology of PFASs analysis and quality assurance in 2016 are given and described in Costantini et al., 2019.^22^ PFASs concentrations were expressed in nanograms per gram of wet weight (ww).

### 2.6 Statistical Analyses

All statistical analyses were performed using R version 4.2.2.^41^ For testosterone and PFTeA values below the LOD, we used the NADA package^42^ to substitute the censored values using the ROS method.^40^ Sperm characteristics may vary according to when sperm is produced relative to when there are used by the females, i.e., the laying of the eggs.^43,44^ Additionally, the sperm velocity and percentage of motile sperm are expected to decrease with the time elapsed from sperm collection to the recording of sperm movement. Due to our relatively small sample size, we chose not to include the duration between sperm collection and the laying of the first egg (days) as well as the time elapsed from sperm collection to sperm video recording (seconds) as covariables in multivariable models. Instead, we first explored whether and how these two variables were correlated with parameters of sperm quality and then decided about their inclusion or not in further models. The laying date was unknown for three males in 2016 and nine males in 2017 and was imputed with the median laying date in each year.

First, we examined the relationship between each PFAS and our four parameters of sperm quality. To this aim, we first examined whether and how the concentrations of PFOSlin, PFNA, PFDcA, PFUnA, PFDoA, PFTrA, PFTeA, ∑PFCAs, and ∑PFASs were correlated within individuals and whether and how sperm parameters were correlated within individuals. Second, we examined potential associations between PFAS concentrations and each of the sperm quality parameters. Third, as an attempt to identify potential underlying physiological pathways, and only for the sperm parameters showing a relationship with one or several PFAS concentrations, we examined (1) the relationships between the sperm quality parameter(s) of interest and blood levels of corticosterone, testosterone, and LH, and (2) the relationships between these hormone levels that showed correlation with sperm parameters and the PFASs that were correlated with the sperm parameter(s) of interest.

We used linear models for all of the analyses. We standardized (centered and scaled to 1 SD) PFASs and hormone concentrations by year to allow meaningful interpretation of their potential relationships with each other and with sperm parameters. The percentage of abnormal sperm and the percentage of motile sperm were assessed in both 2016 and 2017, and a visual inspection of the data suggested that their relationships with PFAS concentration may differ between years. Therefore, relevant models also included year as an explanatory factor together with its interaction with the PFAS concentration. Diagnostic plots were assessed to test whether the data met the modeling assumptions of linearity and homoscedasticity and normality of the residuals. No variable required transformation to meet modeling assumptions.

Following recommendations of the American Statistical Association,^45^ we do not use an arbitrary threshold (e.g., 0.05) to interpret our results, but instead we use *p* values to assess the strength of the statistical evidence to reject the null hypothesis together with effect sizes and 95% confidence intervals that measure the uncertainty around the point estimates.

## 3 Results

Summary statistics (sample size, mean ± sd, median, minimum, and maximum values) for all PFASs, sperm quality parameters, and hormones in 2016 and 2017 are presented in Tables S1 and S2.

### 3.1 Correlations within PFASs Levels, Sperm Quality Parameters, and Hormones Levels

We observed moderate to strong evidence of positive correlations among the plasma concentrations of shorter-chain PFASs: PFOSlin (C8), PFNA (C9), PFDcA (C10), PFUnA (C11), and PFDoA (C12). However, the longer-chain PFASs, PFTrA (C13), and PFTeA (C14), showed little-to-no correlation with other compounds (Figure S1).

We found no evidence for correlations among sperm quality parameters, including the percentage of abnormal sperm, the percentage of motile sperm, VCL, and intramale variation in sperm length (Table S3). Similarly, no correlations were observed among the blood concentrations of corticosterone, testosterone, and LH (Table S4).

### 3.2 Correlations Between PFASs Concentrations and Sperm Quality Parameters

First, we tested whether each sperm quality parameter was associated with the timing of the first egg laid (in days) and found that only the percentage of motile spermatozoa depended on the date of the first egg laid, being higher when sperm collection occurred well before egg-laying and decreasing as the time of egg-laying approached (Table S5). Consequently, we included the timing of the first egg laid as a covariable only in the model that had the percentage of motile spermatozoa as the response variable. We also tested whether the percentages of motile spermatozoa and VCL were related to the time (in seconds) from sperm collection to video recording and did not find any correlations. Therefore, we did not include this parameter as a covariable in the models.

**Figure 1:**
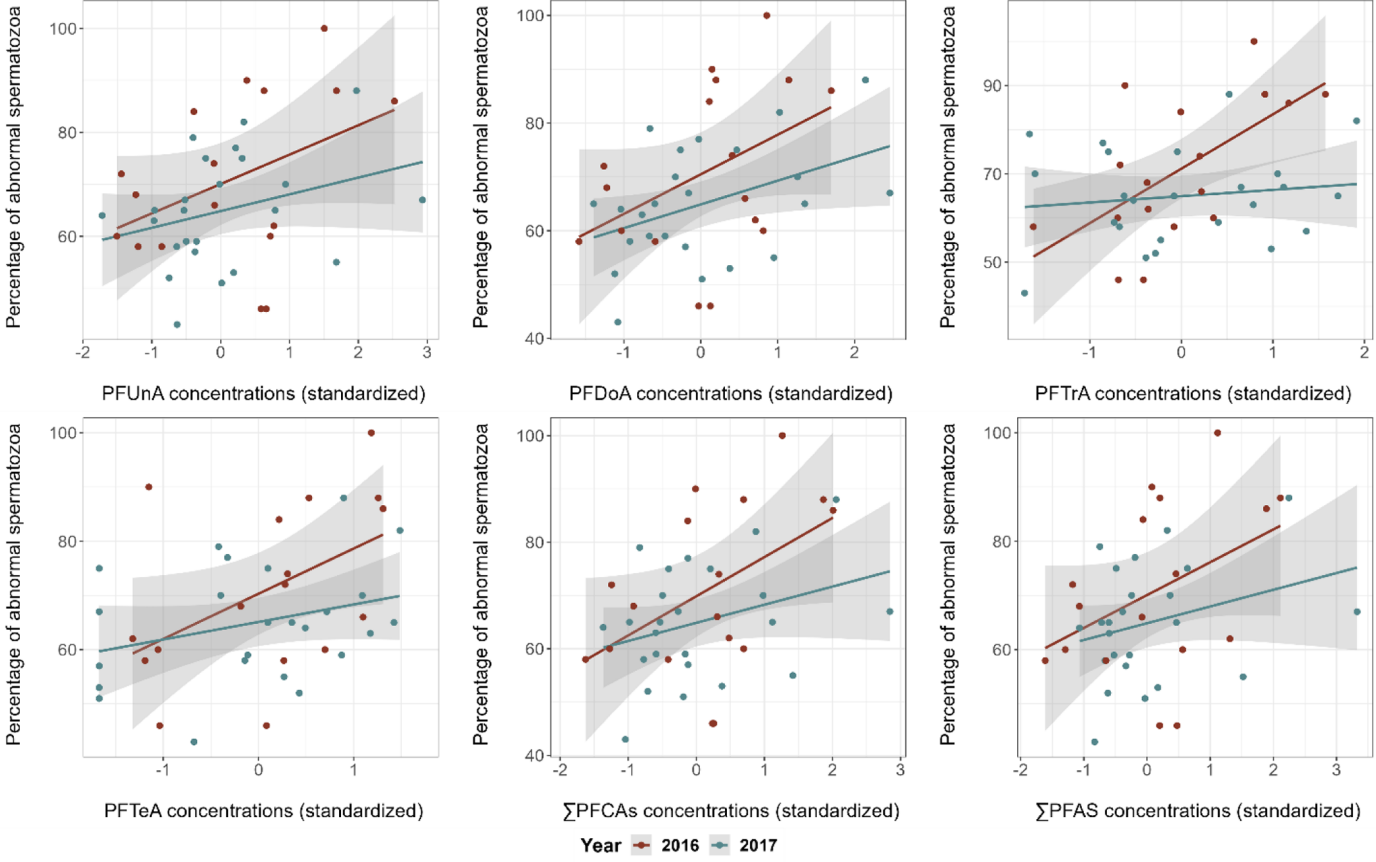
Percentage of abnormal spermatozoa in relation to the plasma concentrations of each PFAS compounds, ∑PFCAs and ∑PFASs (standardized) in 2016 and 2017 for which there is statistical evidence of a correlation in Black-legged kittiwakes (*Rissa tridactyla*).

**Figure 2:**
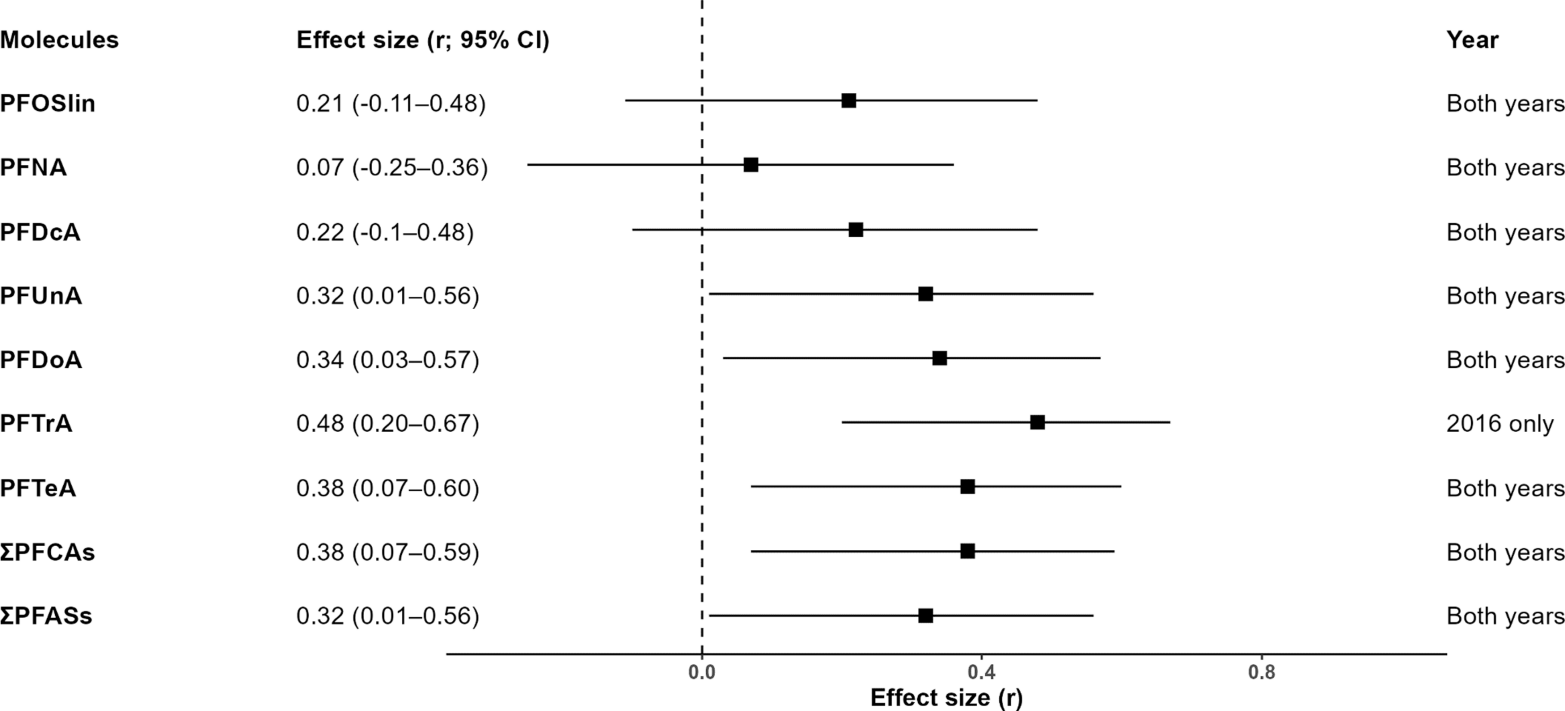
Effect sizes (*r*) along with their 95% confidence intervals (CI) of the relationship between the concentration of each PFAS compound, ∑PFCAs, and ∑PFASs with the percentage of abnormal spermatozoa in Black-legged kittiwakes (*Rissa tridactyla*). The “Year” column indicates whether the effect size *r* and associated CI are related to a single or both years. The PFAS compounds are organized in ascending order based on their carbon chain length (C8 to C14).

We found weak to strong statistical evidence that the percentage of abnormal spermatozoa was positively correlated with blood levels of PFUnA, PFDoA, and PFTeA in all years, and with blood level of PFTrA in 2016 only (Table 1, Figures 1 and 2). There was also weak to moderate evidence that higher ∑PFCAs and ∑PFASs were associated with increased percentages of abnormal sperm (Table 1, Figures 1 and 2). No correlations were found between other sperm quality parameters (i.e., percentage of motile spermatozoa, VCL, and intraejaculate variation in total sperm length) and any of the PFAS concentrations (Tables S6–S8).

**Table 1:** Summary of the Linear Models Examining the Relationship Between the Percentage of Abnormal Spermatozoa and Each PFASs Compound, ∑PFCAs, and ∑PFASs in Black-Legged Kittiwakes (*Rissa tridactyla*)^a^^b^

Percentage of abnormal spermatozoa			
Predictors	Estimate ± SE	95% CI	*p*
**Intercept (year 2016)**	70.49 ± 3.20	[64.01–76.97]	<0.001
**PFOSlin concentration standardized**	3.95 ± 3.00	[−2.12–10.02]	0.20
**Year 2017**	-5.61 ± 4.17	[−14.06–2.84]	0.19
**Standardized PFOSlin × Year**	-1.27 ± 3.99	[−9.36–6.81]	0.75
**Intercept (year 2016)**	70.43 ± 3.46	[63.42–77.44]	<0.001
**PFNA concentration standardized**	1.33 ± 3.24	[−5.23–7.89]	0.68
**Year 2017**	-5.56 ± 4.40	[−14.48–3.36]	0.21
**Standardized PFNA × Year**	1.74 ± 4.21	[−6.78–10.27]	0.68
**Intercept (year 2016)**	69.62 ± 3.31	[62.91–76.32]	<0.001
**PFDcA concentration standardized**	4.00 ± 2.88	[−1.83–9.82]	0.17
**Year 2017**	-4.76 ± 4.25	[−13.37–3.86]	0.27
**Standardized PFDcA × Year**	-1.30 ± 3.90	[−9.21–6.61]	0.74
**Intercept (year 2016)**	70.06 ± 3.09	[63.81–76.31]	<0.001
**PFUnA concentration standardized**	5.65 ± 2.71	[0.17–11.13]	0.04
**Year 2017**	-5.21 ± 4.02	[−13.35–2.93]	0.20
**Standardized PFUnA × Year**	-2.43 ± 3.71	[−9.95–5.09]	0.52
**Intercept (year 2016)**	70.48 ± 2.99	[64.41–76.55]	<0.001
**PFDoA concentration standardized**	7.35 ± 3.34	[0.59–14.11]	0.03
**Year 2017**	-5.59 ± 3.91	[−13.51–2.33]	0.16
**Standardized PFDoA × Year**	-2.95 ± 4.15	[−11.36–5.46]	0.48
**Intercept (year 2016)**	71.16 ± 2.88	[65.33–76.99]	<0.001
**PFTriA concentration standardized**	12.31 ± 3.68	[4.85–19.78]	0.002
**Year 2017**	-6.22 ± 3.76	[−13.84–1.40]	0.11
**Standardized PFTrA × Year**	-10.88 ± 4.39	[−19.77 – −2.00]	0.02
**Intercept (year 2016)**	70.31 ± 3.00	[64.23–76.39]	<0.001
**PFTeA concentration standardized**	8.35 ± 3.35	[1.57–15.13]	0.02
**Year 2017**	-5.21 ± 3.92	[−13.15–2.73]	0.19
**Standardized PFTeA × Year**	-5.11 ± 4.17	[−13.57–3.35]	0.23
**Intercept (year 2016)**	70.06 ± 3.09	[63.79–76.33]	<0.001
**∑PFCAs concentration standardized**	6.06 ± 2.92	[0.15–11.98]	0.02
**Year 2017**	-5.20 ± 4.03	[−13.36–2.97]	0.21
**Standardized ∑PFCAs × Year**	-2.97 ± 3.87	[−10.81–4.87]	0.34
**Intercept (year 2016)**	69.85 ± 3.02	[63.73–75.97]	<0.001
**∑PFASs concentration standardized**	7.35 ± 2.98	[1.32–13.39]	0.04
**Year 2017**	-4.99 ± 3.93	[−12.95–2.98]	0.21
**Standardized ∑PFASs × Year**	-3.95 ± 3.87	[−11.80–3.90]	0.31

a Year is included as covariable.

b The table shows model estimates ± standard error (Est. ± SE) and associated 95% confidence intervals (CI).

### 3.3 Correlations Between PFASs and Hormones Concentrations

**Figure 3:**
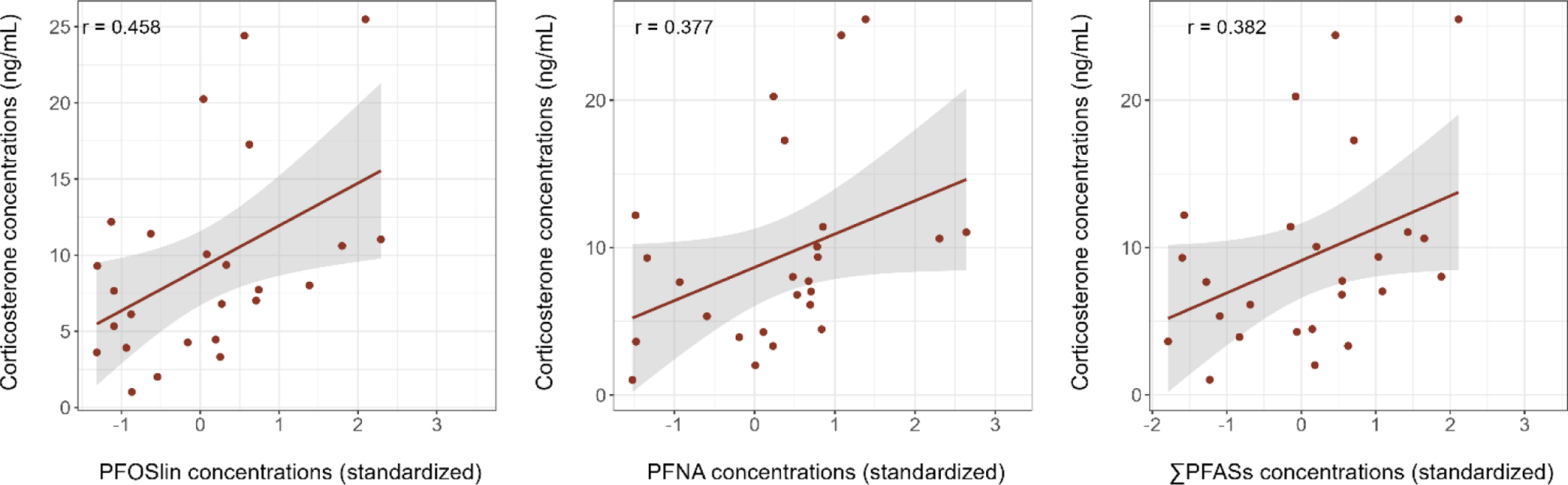
Corticosterone concentration in relation to the plasma concentrations of PFOSlin, PFNA, and ∑PFASs (standardized) in 2016 for which there is statistical evidence of a correlation in Black-legged kittiwakes (*Rissa tridactyla*). Effect sizes (*r*) are added to the corresponding graphs.

We observed weak to moderate statistical evidence in favor of positive relationships between PFOSlin, PFNA concentrations, and corticosterone levels in 2016 (Figure 3, Table 2), with PFUnA and PFDoA also showing weak trends (Table 2). Evidence of a positive correlation between corticosterone concentration and the ∑PFASs was weak (Figure 3, Table 2). No evidence was found of correlations between testosterone or LH levels and any PFAS concentrations (Tables S9 and S10).

**Table 2:** Summary of the Linear Models Examining the Relationships Between Corticosterone Levels and Each PFASs Compounds, ∑PFCAs, and ∑PFASs in Black-Legged Kittiwakes (*Rissa tridactyla*)^a^

Corticosterone (ng/mL)			
Predictors	Estimate ± SE	95% CI	*p*
**Intercept**	9.15 ± 1.18	[6.72–11.58]	<0.001
**PFOSlin concentration standardized**	2.79 ± 1.13	[0.45–5.13]	0.02
**Intercept**	8.66 ± 1.27	[6.04–11.28]	<0.001
**PFNA concentration standardized**	2.26 ± 1.16	[−0.14–4.65]	0.06
**Intercept**	8.83 ± 1.26	[6.23–11.44]	<0.001
**PFDcA concentration standardized**	1.95 ± 1.06	[−0.25–4.15]	0.08
**Intercept**	9.16 ± 1.29	[6.49–11.84]	<0.001
**PFUnA concentration standardized**	1.27 ± 1.14	[−1.09–3.63]	0.28
**Intercept**	9.16 ± 1.24	[6.59–11.73]	<0.001
**PFDoA concentration standardized**	2.14 ± 1.22	[−0.38–4.66]	0.09
**Intercept**	9.39 ± 1.27	[6.77–12.02]	<0.001
**PFTrA concentration standardized**	2.08 ± 1.47	[−0.97–5.13]	0.17
**Intercept**	9.14 ± 1.32	[6.42–11.86]	<0.001
**PFTeA concentration standardized**	1.16 ± 1.35	[−1.64–3.96]	0.40
**Intercept**	9.11 ± 1.27	[6.49–11.73]	<0.001
**∑PFCAs concentration standardized**	1.76 ± 1.16	[−0.65–4.17]	0.14
**Intercept**	9.10 ± 1.23	[6.56–11.64]	<0.001
**∑PFASs concentration standardized**	2.20 ± 1.11	[−0.11–4.50]	0.06

a The table shows model estimates ± standard error (Est. ± SE) and associated 95% confidence intervals (CI)

### 3.4 Correlation Between Corticosterone and Percentage of Abnormal Spermatozoa

Lastly, we investigated the relationship between the concentration of corticosterone and the percentage of abnormal spermatozoa because they both appeared to be correlated with one or several PFAS concentrations. However, we found no evidence of a relationship between corticosterone and percentage of abnormal spermatozoa (Est ± SE = 0.31 ± 0.58, 95% CI = [−0.93–1.55], *p* = 0.60).

## 4 Discussion

To the best of our knowledge, this is the first study investigating the potential association between PFAS exposure and sperm quality in wild birds. Our results indicate positive correlations between plasma PFAS levels and the percentage of abnormal sperm in black-legged kittiwakes from the Norwegian Arctic. The percentage of abnormal sperm is commonly used as a measure of sperm quality and plays a crucial role in determining fertility among numerous species.^46−48^ Our results suggest that among pollutants prevalent in the Arctic, PFAS exposure could be associated with an increase in the amount of abnormal sperm with potential adverse impacts on reproductive outcomes. However, other pollutants, not assessed in our study but known to be present in Arctic seabirds, could also be contributing to the impact on sperm quality, either independently or in synergy with PFAS (e.g.,^49−55^). For instance, although they tend to decrease in Arctic,^56^ polychlorinated biphenyls (PCBs) have been found to adversely affect the reproductive capabilities of adult roosters and American Kestrels, primarily by inhibiting spermatogenesis.^57,58^ Furthermore, mercury and organochlorines, also detected in the Arctic wildlife (e.g.^53,59^) have been correlated to a decrease in sperm quality, including sperm motility and morphology, in both rodents and humans.^60,61^

Given the absence of any observed associations with hormone levels, it is possible that the synthesis of polyunsaturated fatty acids—which are vital for maintaining the stability and fluidity of sperm membranes^62^—may be affected. Interestingly, our study also suggests that the association between PFASs and the percentage of abnormal sperm may be more pronounced with longer-chain PFCAs (e.g., PFUnA, PFDoA, PFTeA), although the relationship of PFTrA with abnormal sperm percentages was observed in 2016 only. In the same kittiwake population, the strength of the correlation between oxidative status markers and PFASs concentrations also increased with the chain length of PFASs.^22^ These observations are in line with the experimental work reporting higher toxicity of PFAS on rat brain cells with increasing carbon chain length.^63^

While research on the reproductive effects of PFAS remains limited in wildlife,^7,23^ there is a growing body of evidence about the adverse effects of some PFAS compounds on spermatic quality in various laboratory mammals and humans. Specifically, the perfluorotetradecanoic acid (PFTeA) has been identified as a disruptor of spermatogenesis in Sprague–Dawley rats.^64^ This disruption was attributed to a decrease in testosterone levels, leading to a marked reduction in the sperm count within the epididymis. Additional research documented the effects of shorter-chain PFAS compounds, including perfluorooctanesulfonic acid (PFOS), perfluorooctanoic acid (PFOA), and perfluorononanoic acid (PFNA), on rodent spermatogenesis, indicating a substantial decline in sperm count. These studies mentioned Sertoli cells, seminiferous tubules, and the epididymis as primary targets of PFAS-induced damage, through direct functional lesions in the testis and inhibition of testosterone synthesis.^65−70^ Further investigations showed that ingestion of PFOS in mice is causing sperm malformation.^71^ In contrast, studies focusing on humans did not observe significant decreases in semen volume or sperm count associated with increases in PFOS, PFOA, and PFNA contamination. Instead, these studies emphasize the influence of PFAS at earlier stages of sperm cell and primary spermatocyte development.^72^ This suggests, at least in humans, that PFAS interfere with the normal developmental and functional processes of these cells rather than causing direct damage to spermatozoa.^72^ This interpretation is corroborated by Ortiz-Sánchez et al., 2022,^73^ who found that PFOS and PFOA can alter the viability and functionality of boar (*Sus scrofa*) spermatozoa during capacitation, potentially due to changes in the plasma membrane that disrupt calcium transport and decrease electrochemical potential, thus impeding spermatozoa response. The above-mentioned discrepancies suggest that the modes of actions of PFASs on spermatic quality may be species-dependent. This stresses the importance to carry more research in birds to unravel how PFAS and other pollutant contamination affects their reproductive health. In addition, most research indicates detrimental effects of PFOS, PFOA, and PFNA on fertility, and we encourage further toxicological studies to focus on the overlooked longer-chain PFCAs (C11–C14). Lastly, PFOS showed the highest concentrations of all of the examined compounds in both 2016 and 2017. However, contrary to what one might expect given its effect on other species, we did not observe any correlation of this compound with sperm quality.

To explore the potential endocrine mechanisms behind the relationship between PFASs and the proportion of abnormal spermatozoa, we investigated the relationships between corticosterone, testosterone, and LH and PFAS concentrations. These hormones are known to influence sperm quality, with corticosterone involved in stress management and reproductive function, testosterone critical for the development of male reproductive tissues and spermatogenesis, and luteinizing hormone essential to trigger testosterone production.^10,28^ Only PFOSlin and PFNA were positively related to corticosterone levels. However, none of the hormones measured in our study were related to the percentage of abnormal quality. Further, no correlation between PFAS and testosterone or LH were found. This suggests two possibilities: (1) in this particular case, any possible detrimental effect of PFAS on spermatic quality may not be triggered by endocrine disruption of these sexual hormones or (2) sperm quality might be compromised by pollutants other than PFASs. The lack of a relation between corticosterone levels and the rate of abnormal spermatozoa further suggests that hormonal pathways may not play major roles in the potential impact of PFAS on sperm quality in this species.

To conclude, our study suggests a positive correlation between PFAS and a higher number of abnormal sperm in Arctic kittiwakes. Yet, we were not able to shed light on potential underlying endocrine disrupting mechanisms. Despite the consistency in the results both in 2016 and 2017, the present correlative approach precludes any firm conclusions about possible causal effects. Furthermore, we cannot preclude the potential influence of other pollutants in the Arctic as contributing factors to the results of this study. We encourage further studies to address similar questions in more free-living species exposed to a wide range of environmental pollutants.

## Supporting Information Available

Additional figures and tables with summary statistics on PFASs and the outputs of additional statistical models (PDF)
