# Supplementary material for: PFAS Exposure is Associated with a Lower Spermatic Quality in an Arctic Seabird
Source: Environ Sci Technol. 2024 Oct 23;58(44):19617–26. doi: 10.1021/acs.est.4c04495 (PMC11542889; doi:10.1021/acs.est.4c04495)
Supplement: Supplementary file 1 — es4c04495_si_001.pdf [file es4c04495_si_001.pdf]

Supporting Information

PFAS exposure is associated with a lower spermatic quality in an Arctic seabird

Ségolène Humann-Guilleminot[1,2*°], Pierre Blévin[3,4°], Geir Wing Gabrielsen[5], Dorte Herzke[6], Vladimir A. Nikiforov[6], William Jouanneau[3], Børge Moe[7], Charline Parenteau[3], Fabrice Helfenstein[2,8], Olivier Chastel[3]

°Co-first author

[1] Department of Environmental Science, Radboud Institute for Biological and Environmental Sciences (RIBES), Faculty of Science, Radboud University, 6500 Nijmegen, The Netherlands

[2] Laboratory of Evolutionary Ecophysiology, Institute of Biology, University of Neuchâtel, 2000 Neuchâtel, Switzerland

[3] Centre d'Etudes Biologiques de Chizé, UMR 7372 CNRS - Université de La Rochelle, 79360 Villiers-en-Bois, France

[4] Akvaplan niva AS, Fram Centre, NO-9296 Tromsø, Norway

[5] Norwegian Polar Institute, Fram Centre, NO-9296 Tromsø, Norway

[6] Norwegian Institute for Air Research, Fram Centre, NO-9296 Tromsø, Norway

[7] Norwegian Institute for Nature Research, NO-7034 Trondheim, Norway

[8] Department of Clinical Research, University of Bern, 3010 Bern, Switzerland

*Correspondence to: segolene.humann@gmail.com

Summary: 11 pages, 1 figure, 10 tables.

1. Results

Table S1. Sample size, median, mean ± standard deviation (sd), minimum and maximum for each PFAS, ∑PFCAs and ∑PFASs (ng/g ww) in 2016 and 2017.

		2016	2017
**PFOSlin**	median	13.89	12.40
	mean ± sd	13.73 ± 6.60	19.14 ± 17.05
	min	5.20	5.84
	max	27.65	79.08
**PFNA**	median	2.36	1.56
	mean ± sd	2.20 ± 0.93	2.02 ± 1.58
	min	0.64	0.60
	max	4.23	7.11
**PFDcA**	median	3.39	2.32
	mean ± sd	3.19 ± 1.39	2.93 ± 1.76
	min	0.98	1.13
	max	5.56	8.34
**PFUnA**	median	11.66	10.62
	mean ± sd	10.69 ± 4.23	12.71 ± 7.37
	min	3.78	0.03
	max	19.55	34.34
**PFDoA**	median	1.81	2.79
	mean ± sd	1.75 ± 0.86	3.03 ± 1.06
	min	0.24	1.55
	max	3.10	5.64
**PFTrA**	median	8.53	11.95
	mean ± sd	8.42 ± 2.75	12.41 ± 2.95
	min	3.29	7.35
	max	13.49	18.06
**PFTeA**	median	1.31	1.50
	mean ± sd	1.21 ± 0.75	1.35 ± 0.78
	min	0.08	0.04
	max	2.42	2.52
**∑PFCAs**	median	28.79	31.16
	mean ± sd	27.46 ± 9.99	34.45 ± 16.55
	min	9.19	16.55
	max	44.64	71.67
**∑PFASs**	median	42.68	44.70
	mean ± sd	41.19 ± 15.86	53.59 ± 29.20
	min	14.39	22.40
	max	69.78	150.75

Table S2. Sample size, median, mean ± standard deviation (sd), minimum and maximum for corticosterone, testosterone, luteinizing hormone (ng/ml) and sperm quality parameters.

		2016	2017
		**Hormones**	
**Corticosterone**	median	7.73	-
	mean ± sd	9.31 ± 6.47	-
	min	1.02	-
	max	25.49	-
**Testosterone**	median	1.08	-
	mean ± sd	1.6 ± 1.53	-
	min	0.19	-
	max	6.64	-
**Luteinizing hormone**	median	5.36	-
	mean ± sd	5.69 ± 2.27	-
	min	2.11	-
	max	13.02	-
		**Sperm quality**	
**Percentage of abnormal sperm**	median	68.00	65.00
	mean ± sd	70.94 ± 16.06	64.96 ± 10.83
	Min	46.00	43.00
	Max	100.00	88.00
**Percentage of motile sperm**	Median	0.19	0.28
	mean ± sd	0.21 ± 0.15	0.33 ± 0.22
	Min	0.02	0.08
	Max	0.58	0.81
**VCL (µm/s)**	Median	141.95	153.12
	mean ± sd	160.69 ± 43.08	153.91 ± 30.14
	Min	108.32	85.24
	Max	264.47	213.88
**Intra-individual variation of total sperm length (µm)**	Median	10.79	-
	mean ± sd	10.15 ± 4.79	-
	min	2.26	-
	max	20.4	-

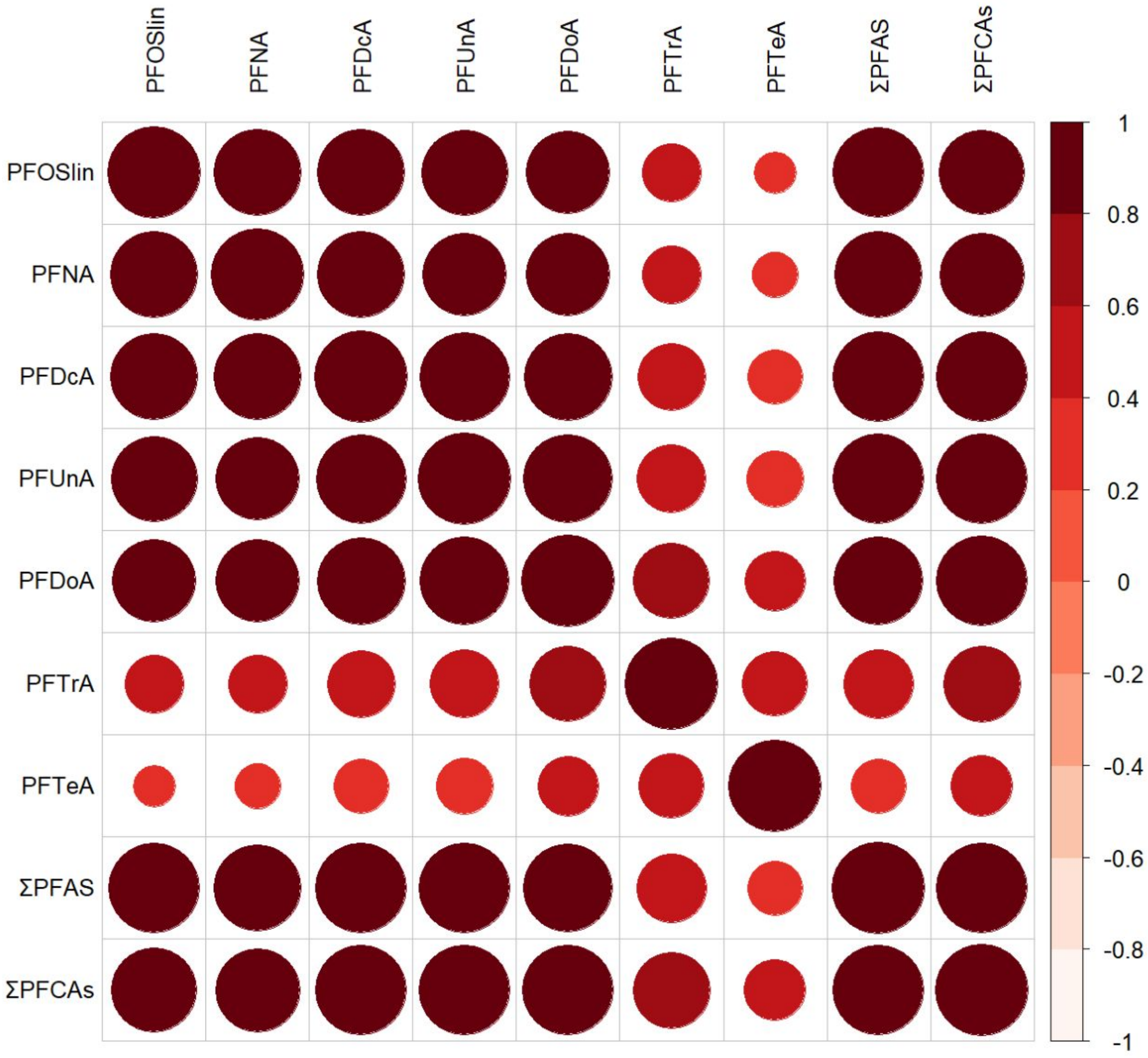

Figure S1. Correlation among PFASs (standardized).

Table S3. Correlation among sperm quality parameters.

	Percentage of abnormal spermatozoa	Percentage of motile spermatozoa	VCL	Variation in total length of spermatozoa
**Percentage of abnormal spermatozoa**	1.00	-0.33	0.05	-0.45
**Percentage of motile spermatozoa**	-0.33	1.00	0.27	-0.40
**VCL**	0.05	0.27	1.00	-0.40
**Variation in total length of spermatozoa**	-0.45	-0.40	-0.40	1.00

Table S4. Correlation among hormone concentrations.

	Testosterone	LH	Corticosterone
**Testosterone**	1.00	0.11	-0.40
**LH**	0.11	1.00	-0.001
**Corticosteron**	-0.40	-0.001	1.00

Table S5. Summary of the linear models examining the relationship between sperm quality parameters and the time from sperm collection to the laying of the first egg.

	Percentage of abnormal spermatozoa		
	**Estimate ± SE**	**95% CI**	**p**
**Intercept (year 2016)**	71.05 ± 5.79	[59.34 – 82.77]	<0.001
**Time elapsed between sperm collection and first egg (days)**	0.27 ± 0.40	[-0.54 – 1.08]	0.51
	**Percentage of motile spermatozoa**		
**Intercept (year 2016)**	0.12 ± 0.07	[-0.02 – 0.26]	0.09
**Time elapsed between sperm collection and first egg (days)**	-0.01 ± 0.00	[-0.02 – -0.00]	0.01
	**VCL**		
**Intercept (year 2016)**	149.75 ± 12.99	[123.50 – 175.99]	<0.001
**Time elapsed between sperm collection and first egg (days)**	-0.53 ± 0.92	[-2.39 – 1.32]	0.57
	**Variation in total length of spermatozoa**		
**Intercept (year 2016)**	10.68 ± 2.28	[5.87 – 15.49]	<0.001
**Time elapsed between sperm collection and first egg (days)**	0.04 ± 0.21	[-0.41 – 0.48]	0.86

Table S6. Summary of the linear model examining the relationship between the percentage of motile spermatozoa with each PFASs, sum of PFCAs, sum of PFASs and year and time elapsed between sperm collection and first egg as covariables. The table shows model estimates ± standard error (Est. ± s.e.) and associated 95% confidence intervals (C.I). Weak statistical evidence in italic.

	Percentage of motile spermatozoa		
**Predictors**	**Estimate ± SE**	**95% CI**	**p**
**Intercept**	0.13 ± 0.07	[-0.02 – 0.27]	0.08
**Time elapsed between sperm collection and first egg (days)**	-0.01 ± 0.01	[-0.02 – 0.00]	0.12
**PFOSlin concentration standardized**	-0.02 ± 0.04	[-0.11 – 0.06]	0.62
**Year 2017**	0.04 ± 0.08	[-0.12 – 0.20]	0.60
**Standardized PFOSlin x Year**	0 ± 0.06	[-0.12 – 0.12]	0.98
**Intercept (year 2016)**	0.12 ± 0.07	[-0.02 – 0.27]	0.10
**Time elapsed between sperm collection and first egg (days)**	-0.01 ± 0.01	[-0.02 – 0.00]	0.10
**PFNA concentration standardized**	-0.01 ± 0.04	[-0.09 – 0.07]	0.90
**Year 2017**	0.04 ± 0.08	[-0.12 – 0.19]	0.65
**Standardized PFNA x Year**	-0.01 ± 0.06	[-0.13 – 0.10]	0.80
**Intercept (year 2016)**	0.13 ± 0.07	[-0.02 – 0.27]	0.09
**Time elapsed between sperm collection and first egg (days)**	-0.01 ± 0.01	[-0.02 – 0.00]	0.11
**PFDcA concentration standardized**	-0.02 ± 0.04	[-0.11 – 0.06]	0.59
**Year 2017**	0.04 ± 0.08	[-0.12 – 0.20]	0.62
**Standardized PFDcA x Year**	0.02 ± 0.06	[-0.10 – 0.13]	0.75
**Intercept (year 2016)**	0.13 ± 0.07	[-0.02 – 0.27]	0.08
**Time elapsed between sperm collection and first egg (days)**	-0.01 ± 0.01	[-0.02 – 0.00]	0.14
**PFUnA concentration standardized**	-0.03 ± 0.04	[-0.13 – 0.06]	0.45
**Year 2017**	0.05 ± 0.08	[-0.11 – 0.21]	0.55
**Standardized PFUnA x Year**	0.03 ± 0.06	[-0.09 – 0.16]	0.58
**Intercept (year 2016)**	0.13 ± 0.07	[-0.02 – 0.27]	0.09
**Time elapsed between sperm collection and first egg (days)**	-0.01 ± 0.01	[-0.02 – 0.00]	0.12
**PFDoA concentration standardized**	-0.02 ± 0.05	[-0.12 – 0.07]	0.62
**Year 2017**	0.05 ± 0.08	[-0.11 – 0.21]	0.56
**Standardized PFDoA x Year**	0.04 ± 0.06	[-0.09 – 0.17]	0.56
**Intercept (year 2016)**	0.13 ± 0.07	[-0.01 – 0.26]	0.06
**Time elapsed between sperm collection and first egg (days)**	-0.01 ± 0.01	[-0.02 – 0.00]	0.09
**PFTrA concentration standardized**	0.00 ± 0.05	[-0.11 – 0.11]	0.97
**Year 2017**	0.04 ± 0.07	[-0.11 – 0.19]	0.57
**Standardized PFTiA x Year**	0.09 ± 0.06	[-0.04 – 0.22]	0.19
**Intercept (year 2016)**	0.12 ± 0.07	[-0.02 – 0.27]	0.09
**Time elapsed between sperm collection and first egg (days)**	-0.01 ± 0.01	[-0.02 – 0.00]	0.11
**PFTeA concentration standardized**	0.02 ± 0.05	[-0.07 – 0.12]	0.62
**Year 2017**	0.04 ± 0.08	[-0.12 – 0.20]	0.64
**Standardized PFTeA x Year**	-0.04 ± 0.06	[-0.16 – 0.09]	0.56
**Intercept (year 2016)**	0.13 ± 0.07	[-0.02 – 0.27]	0.08

**Time elapsed between sperm collection and first egg (days)**	-0.01 ± 0.01	[-0.02 – 0.00]	0.13
**∑PFCAs concentration standardized**	-0.02 ± 0.04	[-0.11 – 0.07]	0.64
**Year 2017**	0.05 ± 0.08	[-0.11 – 0.20]	0.56
**Standardized ∑PFCAs x Year**	0.04 ± 0.06	[-0.08 – 0.16]	0.56
**Intercept (year 2016)**	0.13 ± 0.07	[-0.02 – 0.27]	0.09
**Time elapsed between sperm collection and first egg (days)**	-0.01 ± 0.01	[-0.02 – 0.00]	0.12
**∑PFASs concentration standardized**	-0.02 ± 0.04	[-0.11 – 0.07]	0.65
**Year 2017**	0.04 ± 0.08	[-0.12 – 0.20]	0.59
**Standardized ∑PFCAs x Year**	0.01 ± 0.06	[-0.10 – 0.13]	0.81

Table S7. Summary of the linear model examining the relationship between VCL (µm/s) with each PFASs, sum of PFCAs, sum of PFASs and year as covariable. The table shows model estimates ± standard error (Est. ± s.e.) and associated 95% confidence intervals (C.I). Moderates to strong statistical evidence are in bold.

	VCL (µm/s)		
**Predictors**	**Estimate ± SE**	**95% CI**	**p**
**Intercept (year 2016)**	161.03 ± 8.76	[143.33 – 178.74]	<0.001
**PFOSlin concentration standardized**	-5.92 ± 7.57	[-21.23 – 9.38]	0.44
**Year 2017**	-7.13 ± 11.25	[-29.89 – 15.64]	0.53
**Standardized PFOSlin x Year**	-0.16 ± 10.45	[-21.30 – 20.98]	0.99
**Intercept (year 2016)**	161.33 ± 9.04	[143.04 – 179.61]	<0.001
**PFNA concentration standardized**	-2.29 ± 7.42	[-17.31 – 12.73]	0.76
**Year 2017**	-7.42 ± 11.51	[-30.69 – 15.85]	0.52
**Standardized PFNA x Year**	-3.84 ± 10.38	[-24.84 – 17.16]	0.71
**Intercept (year 2016)**	160.69 ± 8.84	[142.80 – 178.58]	<0.001
**PFDcA concentration standardized**	-0.04 ± 7.67	[-15.57 – 15.48]	1.00
**Year 2017**	-6.78 ± 11.37	[-29.77 – 16.21]	0.55
**Standardized PFDcA x Year**	-5.21 ± 10.58	[-26.61 – 16.19]	0.63
**Intercept (year 2016)**	160.25 ± 8.76	[142.53 – 177.97]	<0.001
**PFUnA concentration standardized**	-8.3 ± 8.24	[-24.97 – 8.37]	0.32
**Year 2017**	-6.34 ± 11.26	[-29.12 – 16.44]	0.58
**Standardized PFUnA x Year**	4.71 ± 10.95	[-17.44 – 26.87]	0.67
**Intercept (year 2016)**	160.09 ± 8.93	[142.04 – 178.15]	<0.001
**PFDoA concentration standardized**	-4.83 ± 9.16	[-23.36 – 13.69]	0.60
**Year 2017**	-6.18 ± 11.44	[-29.33 – 16.96]	0.60
**Standardized PFDoA x Year**	6.37 ± 11.71	[-17.33 – 30.06]	0.60
**Intercept (year 2016)**	160.93 ± 8.7	[143.34 – 178.52]	<0.001
**PFTrA concentration standardized**	1.93 ± 10.41	[-19.12 – 22.98]	0.85
**Year 2017**	-7.02 ± 11.13	[-29.54 – 15.49]	0.53
**Standardized PFTrA x Year**	9.71 ± 12.59	[-15.76 – 35.18]	0.45
**Intercept (year 2016)**	160.92 ± 8.82	[143.09 – 178.76]	<0.001
**PFTeA concentration standardized**	5.82 ± 9.09	[-12.56 – 24.20]	0.52
**Year 2017**	-7.01 ± 11.34	[-29.94 – 15.92]	0.54
**Standardized PFTeA x Year**	-1.79 ± 11.63	[-25.32 – 21.74]	0.88
**Intercept (year 2016)**	160.53 ± 8.88	[142.57 – 178.50]	<0.001
**∑PFCAs concentration standardized**	-3.35 ± 8.55	[-20.64 – 13.94]	0.70
**Year 2017**	-6.63 ± 11.42	[-29.72 – 16.47]	0.56
**Standardized ∑PFCAs x Year**	2.88 ± 11.25	[-19.89 – 25.64]	0.80
**Intercept (year 2016)**	160.67 ± 8.82	[142.82 – 178.52]	<0.001
**∑PFASs concentration standardized**	-4.57 ± 7.85	[-20.44 – 11.30]	0.56
**Year 2017**	-6.76 ± 11.35	[-29.71 – 16.19]	0.55
**Standardized ∑PFCAs x Year**	0.81 ± 10.70	[-20.84 – 22.45]	0.94

Table S8. Summary of the linear model examining the relationship between variation (standard deviation) in total length of spermatozoa (µm) with each PFASs, sum of PFCAs, sum of PFASs in 2016 only. The table shows model estimates ± standard error (Est. ± s.e.) and associated 95% confidence intervals (C.I). Moderates to strong statistical evidence are in bold, weak statistical evidence in italic.

	Variation in total length of spermatozoa (µm)		
**Predictors**	**Estimate ± SE**	**95% CI**	**p**
**Intercept**	9.96 ± 1.13	[7.60 – 12.33]	<0.001
**PFOSlin concentration standardized**	0.66 ± 1.05	[-1.56 – 2.87]	0.54
**Intercept**	7.74 ± 2.9	[1.65 – 13.84]	0.016
**PFNA concentration standardized**	0.00 ± 0.00	[-0.00 – 0.00]	0.38
**Intercept**	9.44 ± 3.04	[3.07 – 15.82]	0.006
**PFDcA concentration standardized**	0.00 ± 0.00	[-0.00 – 0.00]	0.81
**Intercept**	10.17 ± 3.29	[3.26 – 17.09]	0.006
**PFUnA concentration standardized**	0.00 ± 0.00	[-0.00 – 0.00]	0.10
**Intercept**	11.14 ± 2.82	[5.21 – 17.07]	0.001
**PFDoA concentration standardized**	0.00 ± 0.00	[-0.00 – 0.00]	0.78
**Intercept**	15.07 ± 3.46	[7.79 – 22.34]	<0.001
**PFTrA concentration standardized**	0.00 ± 0.00	[-0.00 – 0.00]	0.15
**Intercept**	13.45 ± 1.94	[9.37 – 17.52]	<0.001
**PFTeA concentration standardized**	-0.00 ± 0.00	[-0.01 – 0.00]	0.06
**Intercept**	10.29 ± 1.14	[7.90 – 12.69]	<0.001
**∑PFCAs concentration standardized**	-0.46 ± 1.02	[-2.59 – 1.68]	0.66
**Intercept**	10.16 ± 1.15	[7.75 – 12.57]	<0.001
**∑PFASs concentration standardized**	-0.03 ± 1.01	[-2.16 – 2.11]	0.98

Table S9. Summary of the linear model examining the relationship between testosterone concentrations (ng/ml) with each PFASs, sum of PFCAs, sum of PFASs in 2016 only. The table shows model estimates ± standard error (Est. ± s.e.) and associated 95% confidence intervals (C.I). Moderates to strong statistical evidence are in bold.

	Testosterone (ng/ml)		
**Predictors**	**Estimate ± SE**	**95% CI**	**p**
**Intercept**	1.62 ± 0.31	[0.99 – 2.26]	<0.001
**PFOSlin concentration standardized**	-0.33 ± 0.3	[-0.94 – 0.28]	0.27
**Intercept**	1.67 ± 0.32	[1.00 – 2.34]	<0.001
**PFNA concentration standardized**	-0.23 ± 0.29	[-0.84 – 0.38]	0.44
**Intercept**	1.65 ± 0.32	[0.99 – 2.31]	<0.001
**PFDcA concentration standardized**	-0.19 ± 0.27	[-0.74 – 0.37]	0.49
**Intercept**	1.62 ± 0.32	[0.97 – 2.27]	<0.001
**PFUnA concentration standardized**	-0.14 ± 0.28	[-0.71 – 0.44]	0.63
**Intercept**	1.62 ± 0.31	[0.97 – 2.27]	<0.001
**PFDoA concentration standardized**	-0.18 ± 0.31	[-0.82 – 0.45]	0.56
**Intercept**	1.62 ± 0.31	[0.98 – 2.27]	<0.001
**PFTrA concentration standardized**	0.10 ± 0.36	[-0.65 – 0.85]	0.79
**Intercept**	1.56 ± 0.30	[0.93 – 2.18]	<0.001
**PFTeA concentration standardized**	0.43 ± 0.31	[-0.21 – 1.08]	0.17
**Intercept**	1.63 ± 0.31	[0.98 – 2.28]	<0.001
**∑PFCAs concentration standardized**	-0.07 ± 0.29	[-0.66 – 0.53]	0.82
**Intercept**	1.62 ± 0.31	[0.98 – 2.27]	<0.001
**∑PFASs concentration standardized**	-0.2 ± 0.28	[-0.79 – 0.38]	0.55

Table S10. Summary of the linear model examining the relationship between luteinizing hormone concentrations (ng/ml) with each PFASs, sum of PFCAs, sum of PFASs in 2016 only. The table shows model estimates ± standard error (Est. ± s.e.) and associated 95% confidence intervals (C.I). Moderates to strong statistical evidence are in bold.

	**Luteinizing hormone (ng/ml)**		
**Predictors**	**Estimate ± SE**	**95% CI**	**p**
**Intercept**	5.71 ± 0.46	[4.76 – 6.66]	<0.001
**PFOSlin concentration standardized**	-0.31 ± 0.44	[-1.23 – 0.60]	0.49
**Intercept**	5.73 ± 0.48	[4.74 – 6.73]	<0.001
**PFNA concentration standardized**	-0.15 ± 0.44	[-1.05 – 0.76]	0.74
**Intercept**	5.73 ± 0.47	[4.75 – 6.71]	<0.001
**PFDcA concentration standardized**	-0.16 ± 0.4	[-0.99 – 0.66]	0.69
**Intercept**	5.72 ± 0.46	[4.76 – 6.68]	<0.001
**PFUnA concentration standardized**	-0.26 ± 0.41	[-1.10 – 0.59]	0.54
**Intercept**	5.71 ± 0.46	[4.76 – 6.67]	<0.001
**PFDoA concentration standardized**	-0.27 ± 0.45	[-1.20 – 0.67]	0.56
**Intercept**	5.71 ± 0.46	[4.76 – 6.66]	<0.001
**PFTrA concentration standardized**	0.41 ± 0.53	[-0.69 – 1.51]	0.45
**Intercept**	5.58 ± 0.45	[4.66 – 6.51]	<0.001
**PFTeA concentration standardized**	0.72 ± 0.46	[-0.24 – 1.68]	0.14
**Intercept**	5.70 ± 0.47	[4.74 – 6.66]	<0.001
**∑PFCAs concentration standardized**	-0.09 ± 0.43	[-0.97 – 0.79]	0.84
**Intercept**	5.71 ± 0.46	[4.75 – 6.67]	<0.001
**∑PFASs concentration standardized**	-0.18 ± 0.42	[-1.05 – 0.69]	0.68
